# Biphasic and Stage-Associated Expression of CPEB4 in Hepatocellular Carcinoma

**DOI:** 10.1371/journal.pone.0155025

**Published:** 2016-05-09

**Authors:** Li-Yun Tsai, Yu-Wei Chang, Ming-Che Lee, Ying-Chen Chang, Pei-Ing Hwang, Yi-Shuian Huang, Ching-Feng Cheng

**Affiliations:** 1 Department of Medical Research, Buddhist Tzu Chi General Hospital, Hualien, Taiwan; 2 Institute of Biomedical Sciences, Academia Sinica, Taipei, Taiwan; 3 Department of Surgery, Buddhist Tzu Chi General Hospital and Tzu Chi University, Hualien, Taiwan; 4 General Manager, Mao Ying Genetech Inc., Taipei, Taiwan; 5 Department of Pediatrics, Buddhist Tzu Chi General Hospital and Tzu Chi University, Hualien, Taiwan; Hunter College of The City University of New York, UNITED STATES

## Abstract

Cytoplasmic polyadenylation element binding protein 4 (CPEB4) is a sequence-specific RNA-binding protein and translational regulator, with expression associated with tumor progression. Nevertheless, CPEB4 seems to play paradoxical roles in cancers–an oncogenic promoter in pancreatic ductal adenocarcinoma (PDA) and glioblastomas but a tumor suppressor in hepatocellular carcinoma (HCC). To assess whether CPEB4-regulated carcinogenesis is tissue-specific, we reevaluated the role of CPEB4 in HCC. Although proliferation of hepatocytes appeared normal in CPEB4-knockout (KO) mice after partial hepatectomy, knockdown (KD) of CPEB4 in HepG2 liver cancer cells promoted colony formation *in vitro*. Moreover, the growth of CPEB4-KD cells was accelerated in an *in vivo* xenograft mouse model. In tumorous and adjacent non-tumorous paired liver specimens from 49 HCC patients, the protein level of CPEB4 was significantly upregulated in early-stage HCC but decreased toward late-stage HCC. This finding agrees with changes in CPEB4 mRNA level from analysis of two sets of HCC microarray data from the Gene Expression Omnibus (GEO) database. Taken together, downregulation of CPEB4 likely occurs at the late cancer stage to facilitate HCC progression. Biphasic alteration of CPEB4 expression during HCC progression suggests its complicated role in tumorigenesis.

## Introduction

Many processes involved in tumor development are due to dysregulated gene expression [1]. Transcription factors such as p53, E2F and Twist were found to suppress and/or promote cancers [2–4]. Translational control in carcinogenesis has gained increasing attention because regulated translation of mRNAs is important to keep cell cycle in check [5–7]. Aberrant expression and phosphoryation of some key players in the translational apparatus, such as eukaryotic initiation factor (eIF)4E and eIF4E-binding proteins (4EBPs), enhances the malignancy of cells [8, 9]. Moreover, fragile X mental retardation protein and CPEBs, RNA-binding proteins that govern translation of target-specific RNAs involved in the cell cycle and the epithelial-mesenchymal transition, are often found aberrantly expressed in various cancers [10, 11]. Together with microRNA (miR)-mediated posttranscriptional regulation [12, 13], pleiotropic cascades of dysregulated gene expression eventually transform normal cells to malignant tumors. Thus, significant efforts have been made to identify mRNAs but also non-coding RNAs (e.g., miRs), whose alterations contribute to cancer etiology.

The CPEB family of RNA-binding proteins in vertebrates contains four members, CPEB1, CPEB2, CPEB3 and CPEB4, which regulate translation of target mRNAs in various tissues. All share sequence identity in their carboxy-terminal RNA-binding domain; however, their amino-terminal regulatory domain is highly variable [14] and the mechanisms each uses to control protein synthesis are somewhat different. For example, CPEB1 and CPEB4 regulate translation at initiation [15–17]; whereas CPEB2 interacts with eukaryotic elongation factor (eEF)2 and controls the rate-limiting step of hypoxia-inducible factor (HIF)-1α RNA translation at elongation [18]. Although all CPEBs promote polyadenylation-induced translation of target mRNAs, only the mechanism for CPEB1 has been characterized at the molecular level and their other mechanisms remain to be explored. CPEB1 and CPEB4 regulate mitotic and meiotic cell cycles and mediate malignant transformation [10, 19]. CPEB1 is epigenetically silent in myeloma and gastric cancer and downregulated in ovarian, gastric, colorectal and breast cancers [20–22]. An increase in an exon 4-included CPEB2 isoform enhances anoikis resistance and metastasis of triple negative breast cancer cells [23]. CPEB3 is downregulated in sporadic colorectal cancer and human papillomavirus-positive cervical cancer [24, 25]. CPEB4 is upregulated in pancreatic ductal adenocarcinoma (PDA) and glioblastoma [26] but downregulated in HCC [27]. Regardless of the role as a translational activator or repressor, CPEB1 and CPEB3 might function as a tumor suppressor. Because CPEB4 expression shows opposite expression between PDA and HCC [27], it may promote or suppress carcinogenesis in a tissue-specific and/or stage-dependent manner [14, 28].

In this study, we assessed the role of CPEB4 in stage-defined HCC. CPEB4 deficiency did not affect hepatocyte proliferation during liver regeneration but promoted colony formation of HepG2 cells established from well-differentiated HCC. Moreover, knockdown (KD) of CPEB4 promoted tumorigenesis of HepG2 cells in a subcutaneous-injection xenograph mouse model, which was opposite to findings in RWP-1 cells, derived from moderately to well-differentiated PDA [26]. We examined paired tumorous and non-tumorous specimens from 49 HCC patients by western blot analysis and two microarray datasets of 125 HCC transcriptomes from the GEO database. Analysis of 174 HCC samples at the protein and mRNA levels showed that CPEB4 expression was upregulated in the early stage of HCC but downregulated in the late stage. Thus, CPEB4 may suppress tumorigenicity of HCC only in late stage and likely plays more complicated roles in HCC progression depending on the stage.

## Materials and Methods

### Human HCC Specimens

This study was approved by the Institutional Review Board of Hualien Tzu Chi General Hospital (IRB100-55) with written consents from patients. We obtained 49 primary liver cancer samples with adjacent non-tumorous liver tissues from patients who had undergone curative hepatic resection during 2012–2014 at Hualien Tzu Chi Hospital. The specimens were snap-frozen immediately after surgical removal and stored in liquid nitrogen. HCC stages were classified by the tumor-node-metastasis (TNM) system [29, 30]. The information for all patients is in Table 1.

**Table 1:** Information and analyzed results of 49 HCC patients.

Subject	Age	TNMStage	TumorSize(cm)	AFP	AVI	HBV	HCV	CPEB4(/β-actin)		E-cadherin(/β-actin)	
								Non-T	T	Non-T	T
1	64	1	6.3	3	absent	1	0	0.124	0.413	0.324	0.002
2	54	1	2	<1.3	absent	1	0	0.145	0.251	0.190	0.253
3	51	1	6	3.7	present	1	0	0.238	0.521	0.911	0.020
4	60	1	1	48.6	present	1	0	0.058	0.410	0.864	0.286
5	47	1	1.8	66.8	absent	1	0	0.245	0.302	0.886	0.180
6	60	1	2.5	19.9	absent	1	1	**0.528**	**0.439**	0.468	0.617
7	65	1	2.3	544.3	absent	0	1	0.019	0.250	0.501	0.001
8	71	1	2.3	5	present	1	1	0.482	0.606	0.471	0.727
9	86	1	9.9	7.9	absent	0	1	0.260	0.889	0.732	0.379
10	75	1	3	44.1	present	0	1	0.038	1.079	0.018	0.203
11	64	1	5	3.8	absent	0	1	0.238	0.383	0.733	0.008
12	70	1	2.3	10.7	absent	0	1	0.310	0.643	0.718	0.936
13	51	2	2.3	2.2	present	0	0	0.538	0.691	0.765	0.123
14	86	2	1.5	57.1	present	1	0	0.017	0.164	0.890	0.307
15	38	2	6.5	222.6	present	1	0	0.360	0.870	0.665	0.851
16	52	2	2	2	present	1	0	0.057	0.151	0.542	0.783
17	67	2	3.8	942.4	present	1	0	**0.151**	**0.049**	**0.461**	**0.080**
18	73	2	3.5	5.6	present	0	0	0.164	0.836	0.421	0.408
19	78	2	3	147.7	present	1	0	0.207	0.730	0.625	0.322
20	77	2	2	4.2	present	0	0	0.012	0.280	0.575	0.293
21	68	2	3	76.6	present	1	0	0.064	0.396	0.354	0.630
22	74	2	14	1148.3	absent	0	0	0.003	0.773	0.386	0.351
23	66	2	3	2187.8	present	0	0	0.068	0.611	0.554	0.239
24	57	2	1.8	5.5	absent	1	0	0.069	0.252	0.404	0.354
25	60	2	6.2	31.4	present	0	1	0.456	0.809	0.918	0.043
26	89	2	6.21	324	present	0	1	0.628	0.633	0.512	0.971
27	61	2	3.7	40.9	present	1	1	0.161	0.215	0.998	0.208
28	66	2	2.5	2	present	0	1	0.040	0.067	0.842	0.713
29	70	2	2.4	434.9	present	1	1	0.517	0.563	0.225	0.566
30	62	2	3.2	4.8	present	0	1	0.046	0.957	0.162	0.955
31	46	2	17	242.1	present	0	1	0.080	0.807	0.301	0.292
32	69	2	4.5	99.3	present	0	1	0.212	0.479	0.397	0.175
33	58	2	2.5	15.9	present	0	1	0.146	0.687	0.469	0.089
34	72	2	1.6	18.6	present	0	1	0.243	0.899	0.701	0.667
35	74	2	6	4.3	present	1	1	0.028	0.542	0.268	0.015
36	73	2	4.5	13	present	0	1	**0.608**	**0.504**	0.278	0.771
37	66	3a	10	5.4	present	1	0	**0.291**	**0.153**	**0.297**	**0.043**
38	39	3a	10.5	354.7	present	1	0	0.142	0.412	0.390	0.388
39	74	3a	7	8.2	present	0	1	0.261	0.567	0.571	0.609
40	60	3a	6.5	59.7	present	0	1	**0.169**	**0.101**	0.058	0.106
41	77	3a	9	11.9	present	0	1	0.040	0.092	1.109	0.051
42	82	3b	12	3.9	present	0	0	0.078	0.395	0.940	0.047
43	42	3b	1	31.7	present	1	0	0.062	0.671	0.600	0.013
44	73	3b	15	2076.2	present	0	0	0.365	0.895	0.744	0.376
45	38	3b	10	396966	present	1	0	0.083	0.780	0.261	0.424
46	60	3b	15	10.8	present	1	1	0.204	0.516	2.204	0.178
47	64	**3c**	10	6.3	present	0	1	**0.042**	**0.013**	**0.842**	**0.118**
48	63	**4a**	5	140.1	present	1	0	**0.787**	**0.136**	**0.544**	**0.057**
49	61	**4b**	6	441	present	0	1	**0.081**	**0.019**	**0.880**	**0.156**

HCC tumors were staged using the tumor-node-metastasis (TNM)

AFP, plasma α-fetoprotein (ng/ml)

AVI, Angiolymphatic invasion

HBV, Hepatitis B virus; HCV, Hepatitis C virus (1, infected; 0, not infected)

non-T, non-tumorous tissue; T, tumorous tissue

CPEB4 and E-cadherin levels were normalized with β-actin signal

highlighted in bold: reduced CPEB4 expression/ simultaneous decrease in CPEB4 and E-cad levels in tumorous tissues

underlined: CPEB4 level remained unchanged in tumorous tissues

### Ethics Statement

This study was approved by Institutional Animal Care and Use Committee (IACUC) of Academia Sinica (protocol number: 12-10-413) and compliant with Taiwan Ministry of Science and Technology guidelines for ethical treatment of animals. All experimental protocols were performed in accordance with the guidelines of IACUC. Appropriate anesthesia was applied for partial hepatectomy (PH) and *in vivo* tumor growth assay as described below. All mice were recovered normally after PH and cell injection, so no post-operative analgesic was administered to these animals. Animal health and clinical parameters were monitored 3 days a week during the experiments. The indices of endpoint include: 1) tumor burden is greater than 10% body weight or exceeds 20 mm in dimension, 2) tumor interferes with eating or impairs activity, 3) animals have lost more than 15–20% of their body weight, and 4) animal’s skin and fur appeared discoloration, pallor, sore, wound, alopecia and ruffled. All mice remained in good health during two-month monitoring of tumor growth, so no mice were sacrificed prior to the experimental endpoint. All efforts were made to minimize the number of mice used and their suffering. The mice were euthanized with CO_2_ inhalation prior to tissue isolation.

### Partial Hepatectomy (PH) and *In Vivo* Tumor Growth Assay

Total 12 CPEB4 wild-type (WT) and 12 knockout (KO) mice were used for the PH experiment and 18 severe combined immunodeficiency (SCID) mice were used for tumor growth assay. The mice were housed under a 12-h light/dark cycle in a climate-controlled room with *ad libitum* access to food and water. Generation and characterization of CPEB4 KO mice were described before [31]. CPEB4 WT and KO mice were littermates from heterozygous mating. Once the mouse genotype was determined by PCR as described [31], WT and KO male mice after weaning were housed 4–5 per cage until 2 months old. The mouse body weight was measured right before PH. Mice were anesthetized with 87.5 mg/kg ketamine (Merial Laboratoire) and 12.5 mg/kg xylazine. After midline incision of abdominal skin and muscle, the left lateral and median lobes (~2/3) of the liver were ligated at the base. To prevent a circadian influence on cell cycle, this procedure was conducted on 10 mice of alternate WT or KO genotype during 9:00–12:00. The abdominal wall and skin were then sutured separately [32]. One more pair of WT and KO mice were sham-operated to collect liver tissues. After recovery from surgery, mice were killed at the designated times by CO_2_ inhalation and liver samples were collected for weight measurement, then homogenized for western blot analysis. The same number of mice were used to repeat another round of PH experiment. For tumor growth assay, approximately 10^6^ HepG2 cells, untransfected (mock), control (siCtrl) or CPEB4-KD (siCP4), were subcutaneously injected in 8-week-old SCID mice to evaluate their tumorigenicity. Because one SCID mouse appeared unhealthy and was sacrificed by CO_2_ euthanasia, only 17 mice were used for the experiment. The mice after brief isoflurane anesthesia were injected with siCP4 cells in their right flanks and mock or siCtrl cells in their left flanks. The length and width of palpable tumors was measured use of a vernier caliper at various time points. The tumors from both flanks of mice after CO_2_ euthanasia were isolated 2 months after injection to measure their weight.

### Antibodies and Chemicals

We used antibodies for β-actin (AC-15) and CPEB4 (HPA038394) from Sigma-Aldrich; PCNA (#53764) from AnaSpec; CCNB1 (#4138) from Cell Signaling Technology; and E-cadherin (sc-59905) from Santa Cruz Biotechnology. CPEB4 polyclonal and monoclonal antibodies raised against the N-terminal 427 amino acids (a.a.) of rat CPEB4 were as described [31, 33]. With the exception of the Vectastain Elite ABC kit (cat No. PK-6102, Vector labs), all other chemicals were purchased from Sigma-Aldrich.

### Western Blot Analysis

Liver tissues were homogenized in lysis buffer containing 60 mM Tris-HCl pH 6.8, 2% SDS, 10% glycerol and 1X protease inhibitor cocktail (Roche Diagnostics). The homogenized lysates were microcentrifuged at 14,000 rpm for 10 min at 4°C and the protein concentration of supernatant was determined by a BCA protein assay kit (Pierce). Aliquots of 40 μg protein per sample were separated by SDS-PAGE, followed by electroblotting to polyvinylidene difluoride (PVDF) membranes (Millipore). After 1-h blocking in 5% non-fat dry milk, membranes were incubated overnight with primary antibodies at 4°C. After three washes of Tris-buffered saline and Tween 20 (TBST), membranes were incubated with corresponding horseradish peroxidase-conjugated secondary antibody, goat anti-mouse or goat anti-rabbit IgG antibody (Santa Cruz Biotechnology), for 1 h. Immunoreactive bands were detected by an enhanced chemiluminescence plus kit (Amersham Pharmacia Biotech) and quantified by using ImageJ (National Institutes of Health). The molecular weight marker was from Fermentas (PageRuler prestained protein ladder, SM0671).

### RNA Extraction and Quantitative PCR (qPCR)

Total RNA extracted from cultured cells using TRIzol reagent (Invitrogen) was reverse transcribed by using oligo-dT and ImPromII Reverse Transcriptase (Promega). Quantitative PCR (qPCR) involved use of the Universal Probe Library and Lightcycler 480 system (Roche). The PCR primers used were for CPEB4, 5′- ACAGTGACTTTGTGATGGATGG and 5′-TTATCATCGCAAGCTCCACA; β-actin, 5′-CCAACCGCGAGAAGATGA and 5′-CCAGAGGCGTACAGGGATAG. The data analysis involved the comparative Ct (threshold cycle value) method with β-actin mRNA as the reference.

### Microarray Data Collection and Processing

Microarray studies involved datasets for normal liver and stage-defined HCC samples, GSE6764 [34] and GSE9843 [35–37], respectively, from the GEO database (http://www.ncbi.nlm.nih.gov/geo/), obtained in raw data format (.cel). Both datasets were analyzed on an Affymetrix platform (Human Genome U133 Plus 2.0 Array) with the Affy software package under R programming language from the Bioconductor website. Raw data files (.cel) were preprocessed with the Robust Multichip Average (RMA) algorithm in the Affy package. The raw intensity values were background-corrected, normalized among chips, log2 transformed, then output as.txt files. The log2-transformed intensity values for CPEB4 probe IDs, 224828_at, 224829_at and 224831_at (http://www.affymetrix.com), were grouped by HCC stage.

### Cell Culture and DNA Transfection

Human HCC cell lines, HepG2, Hep3B, SNU387 (from American Type Cell Culture) and Mahlavu cells [38], were obtained from Dr. YS Jou (Academia Sinica). These cells were cultured in high-glucose Dulbecco’s modified Eagle’s medium (DMEM) with 10% fetal bovine serum (FBS) and antibiotics. The pGPU6/GFP/Neo-shCPEB4 or pGPU6/GFP/Neo plasmid was transfected into HepG2 cells by using Lipofectamine 2000 reagent (Invitrogen) according to the manufacturer's instructions. Briefly, 4 μg plasmid DNA was mixed with 12 μl Lipofectamine and incubated for 20 min. The liposome-DNA complex was then added to the 6-cm plate of HepG2 cells seeded on the day before transfection. After 24-h transfection, fresh medium containing 400 μg/ml G418 was replaced. The transfected (~20% transfection efficiency) cells under G418 selection were subcultured at 1:5 ratio when reaching confluency. Stably transformed cells expressing GFP, further selected by the flow sorter (FACSAria II), were amplified to collect sufficient cells for *in vitro* and *in vivo* growth assay.

### Colony Formation Assay

HepG2 cells untransfected (mock) or stably transfected with control (siCtrl) or siCPEB4 plasmid were seeded on 6-well plates at 1,000 cells/well. Fresh medium was replaced 24 h later, then changed every 3 days for 3 weeks. Cell colonies were washed with phosphate-buffered saline (PBS) twice, fixed with 4% formaldehyde for 20 min and permeabilized with methanol for 30 min, then stained with 1:20 modified Giemsa (Sigma). After three washes of PBS to remove excess dye, the number of colonies formed was analyzed by using ImageJ.

### Immunohistochemistry and Imaging Acquisition

Sections of CPEB4-WT and -KO mouse brain and liver tissues were fixed in 4% formaldehyde for 10 min, followed by antigen retrieval in 10 mM sodium citrate buffer, pH 6 at 70°C for 20 min. Unless otherwise specified, all procedures were carried out at room temperature. After two washes of PBS, the samples were permeabilized with 0.2% TritonX-100 in PBS, rinsed with PBS three times, blocked for 1 h in 3% bovine serum albumin (BSA) in PBS, then incubated with anti-CPEB4 antibodies at 4°C overnight. After three washes of PBS, the slices were incubated with biotinylated anti-rabbit IgG at room temperature for 1 h, washed with PBS three times, then incubated with the avidin-biotin complex mixture for 30 min. After three washes with PBS, the slices were developed by adding 3, 3′-diaminobenzidine (DAB) substrate until the appearance of a brownish color and mounted on slides. Images were acquired under a Zeiss Z1 microscopy with a Plan-Apochromat 10X DIC II objective lens.

### Plasmid Construction and Lentivirus Production

The shRNA sequence CTGCCTCATTTGGCGAATA targeted to human CPEB4 mRNA was cloned into the pGPU6/GFP/Neo vector (Invitrogen). The DNA fragments of enhanced green fluorescent protein (EGFP), myc-tagged full length and the C-terminus of rat CPEB4 [33] were cloned to the lentiviral vector, pLL3.7-Syn. Lentiviruses were produced following the procedures described previously [39].

### Lentiviral Infection and Cell Proliferation Assay

Mahlavu cells were subcultured the day before infection and then incubated with lentiviral particles for two days prior to the change of fresh medium on day 3. The infected cells were seeded at 1000 cells/ well in 6-well plates. The fresh medium was changed at the day after seeding and every 3 days later. Before the change of medium, 1X PrestoBlue reagent (Invitrogen) was added to the cells and incubated at 37°C for 1 h prior to fluorescence measurement. After 10 days, cells were fixed for colony formation assay as described above.

## Results

### Normal Liver Regeneration in CPEB4-KO Mice after PH

**Fig 1:**
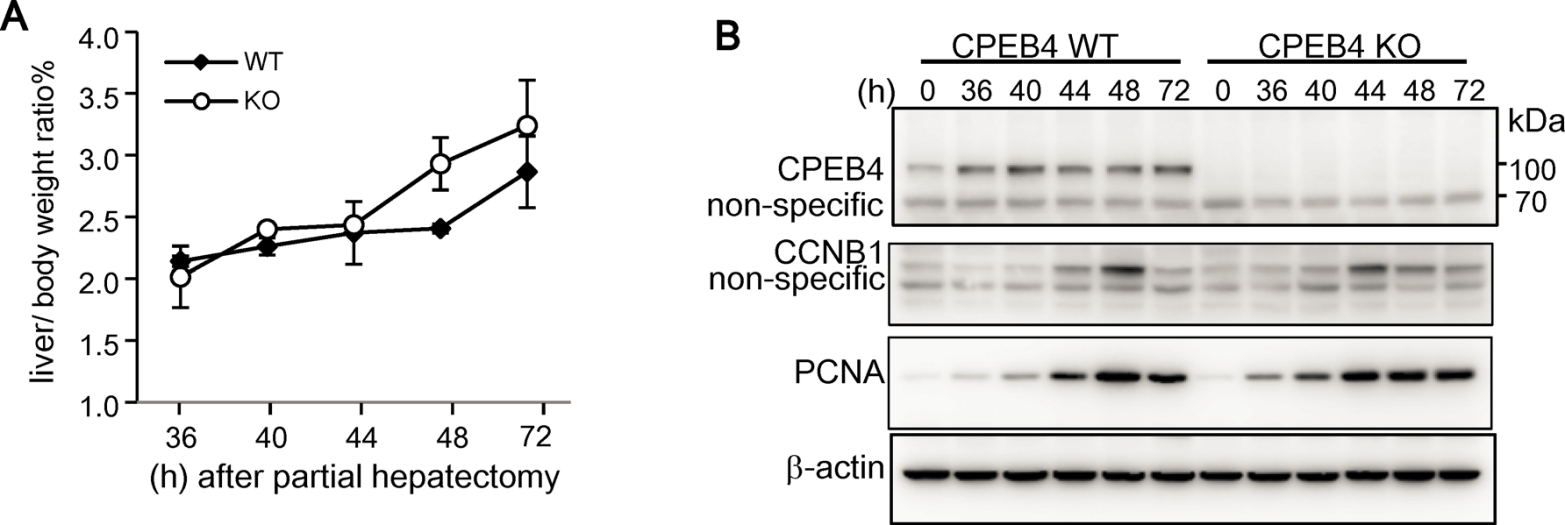
Normal liver regeneration in CPEB4-knockout (KO) mice after partial hepatectomy (PH). (A) The body weight of 2-month-old CPEB4 wild-type (WT) and -KO male littermates was measured, then mice underwent 70% PH. At the designated time after the surgery, livers were isolated for measuring liver weight/body weight. Date are mean ± standard deviation from 2 WT or KO mice per time point. (B) Western blot analysis of CPEB4, cyclin B1 (CCNB1), proliferating cell nuclear antigen (PCNA) and β-actin in liver tissues. The liver tissues at the time-zero-point were collected from sham-operated mice.

CPEB4 is widely expressed in many tissues [31]. CPEB4-KD or overexpression affected the mitotic or meiotic cell cycle in HeLa cells, CD4/CD8-double positive thymocytes and *Xenopus* oocytes [40–42]. We previously generated CPEB4-null mice in a C57BL6 genetic background, which were fertile, with similar growth rate and body weight as their WT littermates [31]. Thus, the loss of CPEB4 may be developmentally compensated. Because CPEB4 is expressed in the adult liver and its downreguation is correlated with HCC progression [27], here, we used PH-induced liver regeneration to examine whether CPEB4 may affect cell cycle reentry of quiescent hepatocytes. Liver regeneration after two-thirds PH is a model system to study cell cycle control in which most remaining hepatocytes escape quiescence to enter the G1 phase of the cell cycle. During the initial 48 h after PH, these hepatic cells enter the S phase and replicate at a relatively synchronous path [43, 44]. CPEB4 -WT and -KO mice underwent PH and their livers were isolated at the designated times after the surgery for weight measurement, expressed as ratio of liver to body weight (Fig 1A), which is about 4–5% in adult mice before PH [45]. CPEB4-KO livers regenerated at a speed similar to WT livers. Hepatic lysates were then examined by western blot analysis of cell-cycle regulatory proteins, including cyclin B1 (CCNB1) for mitosis in G2/M phase and proliferating cell nuclear antigen (PCNA) for DNA replication in S phase as well as CPEB4 (Fig 1B). CPEB4 level remained relatively constant and the expression of CCNB1 and PCNA peaked at about 44–48 h in both WT and KO regenerated livers. Thus, CPEB4-KO mice had no obvious cell cycle defects in normal cells.

### CPEB4 Downregulation Accelerated *In Vitro* and *In Vivo* Growth of HepG2 Cells

**Fig 2:**
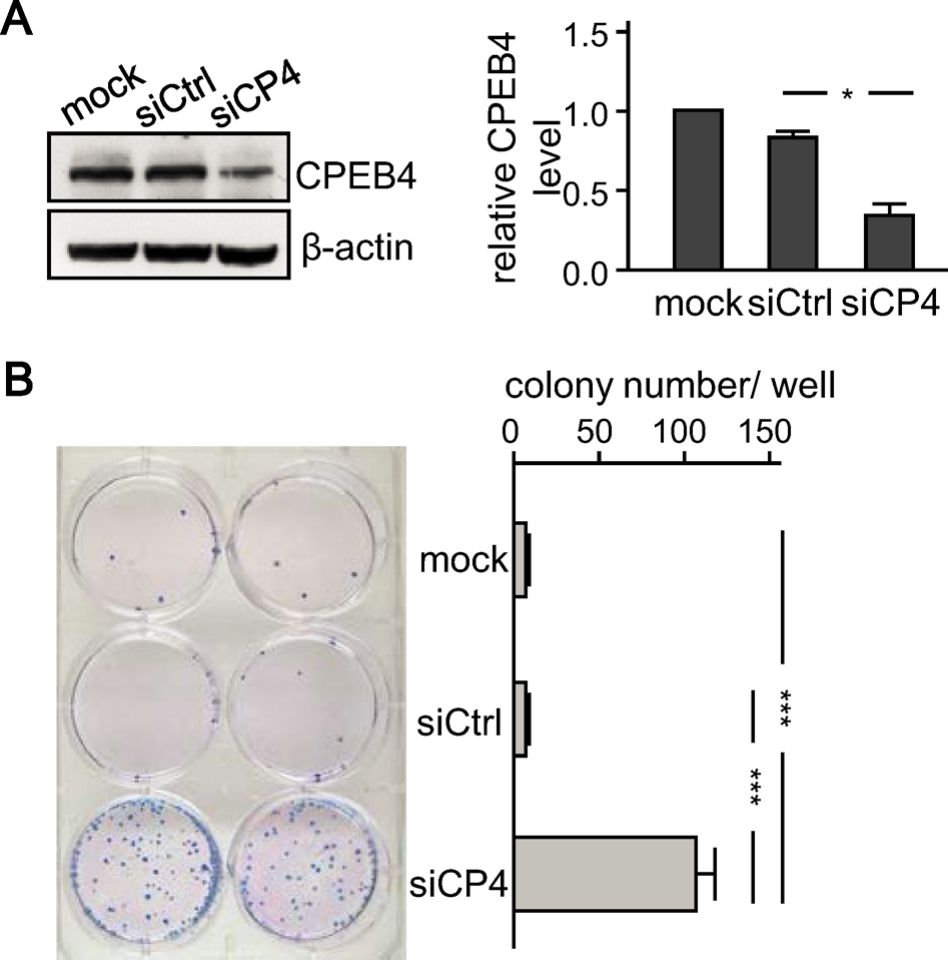
Knockdown of CPEB4 promoted colony formation of HepG2 cells. (A) Immunoblotting of CPEB4 and the loading control β-actin in HepG2 cells stably transfected with the plasmid expressing CPEB4 siRNA (siCP4) or control siRNA (siCtrl) and untransfected (mock) cells. The statistic difference in CPEB4 level from three independent experiments was evaluated by Student’s *t* test, *P < 0.05. (B) Mock, siCtrl and siCP4 HepG2 cells were seeded at low density in 6-well plates and then grew for 3 weeks for colony formation. A representative image is shown on the left. The number of colonies counted in duplicated wells from 2 independent experiments is expressed as mean ± SEM from 4 experiments. ***P < 0.001 by Student’s *t* test.

CPEB4-KD decreased proliferation and colony formation of RWP-1 pancreatic cancer cells *in vitro* and tumorigenesis *in vivo* [26] but increased *in vitro* migration and invasion of SMMC-7721 liver cancer cells [27], which suggests that CPEB4 could promote or suppress tumorogenesis depending on the cancer type. To determine whether CPEB4-KD promotes *in vivo* proliferation of HCC cells, we used HepG2 cells, which express a medium-to-high level of CPEB4 [27], transfected with the plasmid expressing the GFP reporter and CPEB4-KD sequence, which is identical to the validated one used in the previous study [27]. HepG2 cells with CPEB4-KD siRNA (siCP4) or control siRNA (siCtrl) were under G418 selection to remove untransfected cells and then collected for GFP-positive cells by a fluorescence-activated cell sorter. These siCtrl and siCP4 cells (S1 Fig) were amplified and used for the growth assay. CPEB4 protein level was significantly lower in siCP4 than untransfected (mock) and siCtrl cells (Fig 2A). The number of colonies formed was significantly increased in siCP4 than mock or siCtrl HepG2 cells (Fig 2B).

**Fig 3:**
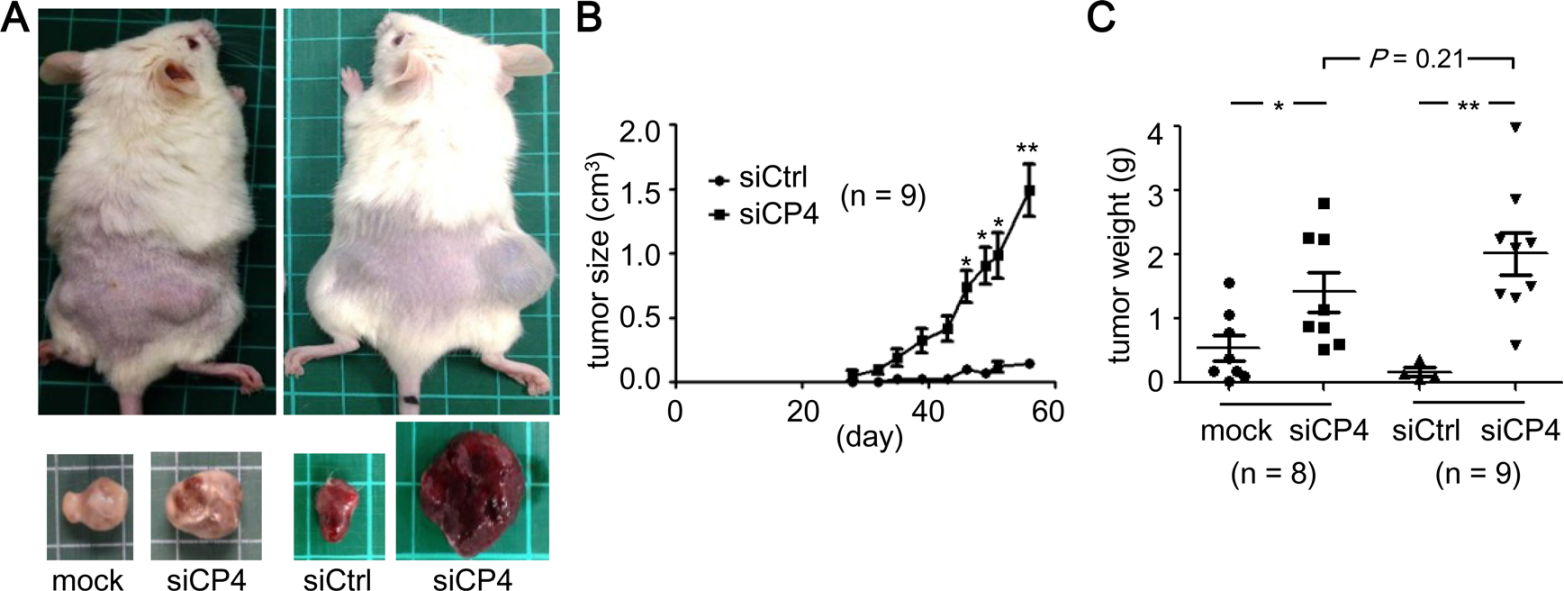
Knockdown of CPEB4 increased tumorigenesis of HepG2 cells in xenograft SCID mice. CPEB4-KD (siCP4) cells along with untransfected (mock) or control (siCtrl) cells were subcutaneously injected in both flanks of SCID mice. (A) Representative images of mice and tumors at 60 days after injection of denoted HepG2 cells. Green line grid, 1 cm. (B) Tumor growth curves. The length and width of palpable tumors in SCID mice initially injected with siCtrl and siCP4 HepG2 cells were measured at the indicated time. Tumor size was calculated as length x (width)^2^/2. (C) Weights of tumors developed in the group of SCID mice injected with mock and siCP4 cells were 0.53 ± 0.19 g and 1.42 ± 0.31 g (n = 8), and siCtrl and siCP4 cells were 0.16 ± 0.06 g and 2.02 ± 0.33 g (n = 9), respectively. Data are mean ± SEM. No statistic difference between the siCP4 tumors isolated from mock and siCtrl groups (*P* = 0.21, unpaired Student’s *t* test). *P < 0.05, **P < 0.01 by Student’s *t* test.

HepG2 cells are not tumorigenic in nude mice, so we then assessed whether CPEB4-KD could promote *in vivo* tumorigenicity of those cells. Severe combined immunodeficiency (SCID) mice were injected subcutaneously with siCP4 cells along with untransfected (mock) or siCtrl cells in both of their flanks (Fig 3A). The volume of subcutaneous tumors was measured and plotted against the time of measurement (Fig 3B). Tumor growth was greater with siCP4 cell injection (Fig 3B, *P* < 0.01, two-way ANOVA). The tumors from both flanks were isolated 2 months after injection to measure their weight. The tumors developed from siCP4 cells weighted significantly more than those from mock or siCtrl cells (Fig 3C). Thus, downregulation of CPEB4 expression promoted *in vitro* and *in vivo* growth of HCC cells.

### Evaluation of Specificity of CPEB4 Antibodies

**Fig 4:**
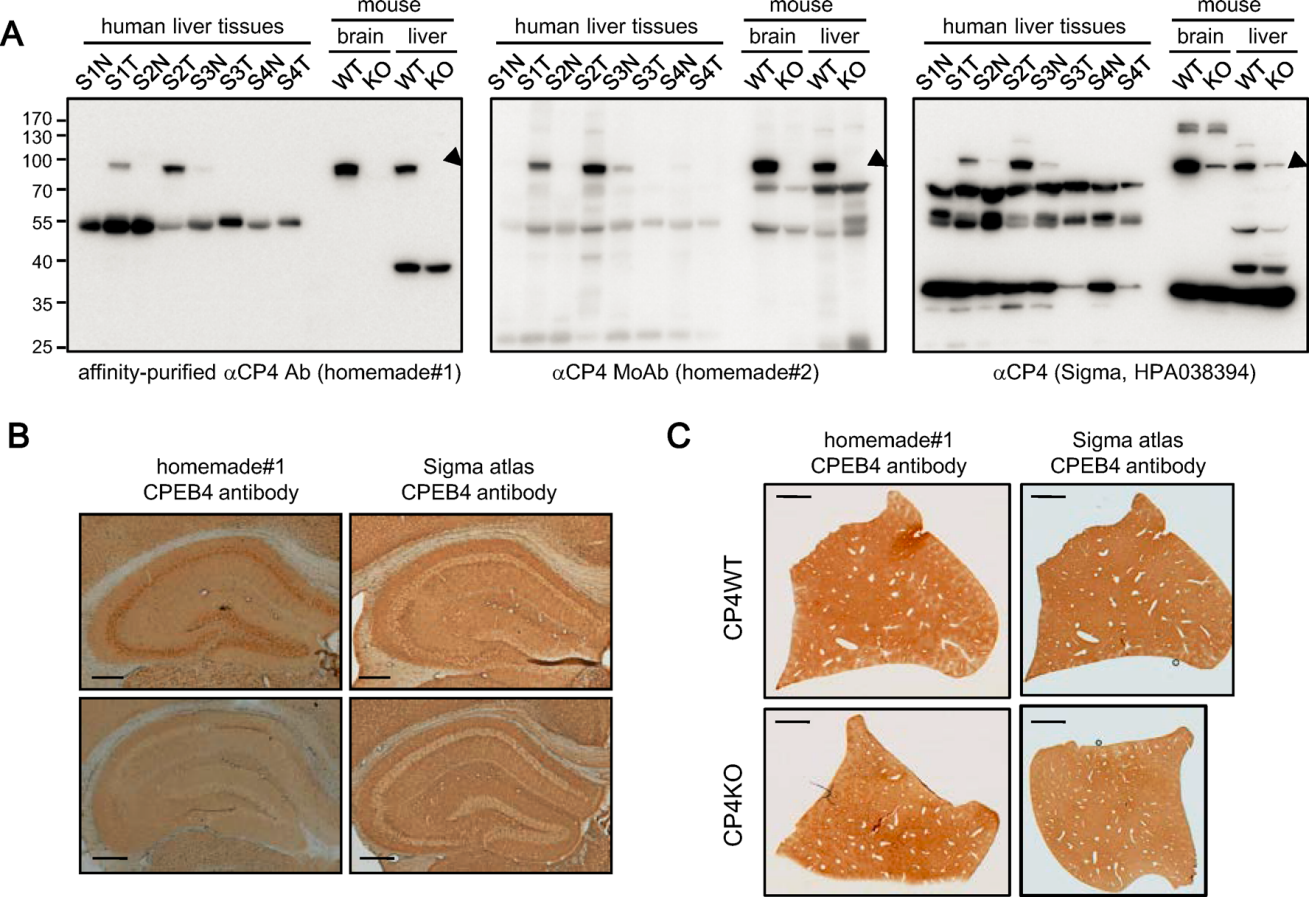
Assessment of specificity of CPEB4 antibodies. (A) Western blot analysis with 2 homemade and one commercial (Sigma) CPEB4 antibody in selected tumorous (T) and adjacent non-tumorous (N) liver samples from HCC patients at different stages (S1-S4) and brain and liver tissues from CPEB4-WT and -KO mice. Arrowheads denote the common band recognized by all 3 antibodies. (B,C) Immunohistochemistry of Homemade#1 and Sigma CPEB4 polyclonal antibodies in (B) brain and (C) liver tissues from CPEB4-WT and -KO mice. Scale bars, 0.25 mm in (B) and 2 mm in (C).

Because we observed no growth defect or spontaneous tumor formation in CPEB4-KO mice, altered CPEB4 expression may affect proliferation of transformed cells but not normal cells. A previous study [26] reported that CPEB4 protein was overexpressed in a large variety of tumors (17 of 20 tumor types in the Human Protein Cancer Atlas, http://www.proteinatals.org/cancer), so we first checked whether liver cancer belongs to three other tumor types. However, immunohistochemistry data with the HPA038394 antibody (Sigma-Aldrich) in the Atlas only indicated that several malignant carcinoids, skin, colorectal and renal cancers along with a few endometrial and pancreatic cancers exhibited moderate cytoplasmic and membranous immunoreactivity. Most cancers were weakly stained or negative for CPEB4. After closely examining the Atlas data, we questioned the specificity of this CPEB4 antibody (HPA038394). First, the major immunoreactive band is about 70 kDa. Second, the immunostaining pattern showed cytoplasmic and Golgi localization. However, both human and mouse CPEB4 of 80 kDa typically migrate to 90–95 kD on SDS-PAGE [26, 31]. Moreover, CPEB4 is primarily localized in the cytoplasm and clustered to RNA-containing stress granules under overexpression, arsenite or heat shock stress [33]. Thus, to the best of our knowledge, the Atlas data related to CPEB4 protein expression is not accurate. Nevertheless, this antibody was used previously to immunostain clinical HCC specimens [27]. Before determining the amount of CPEB4 in our HCC samples, we first used western blot analysis to compare the specificity of the HPA038394 antibody and our polyclonal and monoclonal (Mo) CPEB4 antibodies [31, 33] with selected tumorous (T) and adjacent non-tumorous (N) liver samples from patients with HCC at different stages (S1-S4) as well as brain and liver tissues from CPEB4-WT and -KO mice (Fig 4A). The affinity-purified polyclonal CPEB4 antibody (homemade#1) was specific in both immunoblotting (Fig 4A) and immunostaining assays (Fig 4B) in the brain, as judged by the complete absence of immunoreactive signals in the CPEB4-KO brain, but not in liver tissue (Fig 4A and 4C). The only common band recognized by all three antibodies migrated at about 90–95 kDa on SDS-PAGE (Fig 4A, arrowheads). The HPA038394 antibody, used in the previous HCC study [27] and in the Atlas, detected many non-specific bands of strong signal intensity across human and mouse, brain and liver tissues (Fig 4A). Similarly, this antibody did not specifically detect CPEB4 on immunohistochemistry assay (Fig 4B and 4C) because the immunostained signals were comparable between WT and KO tissues.

### CPEB4 Expression Is Upregulated in Early-Stage but Decreased in Late-Stage HCC

**Fig 5:**
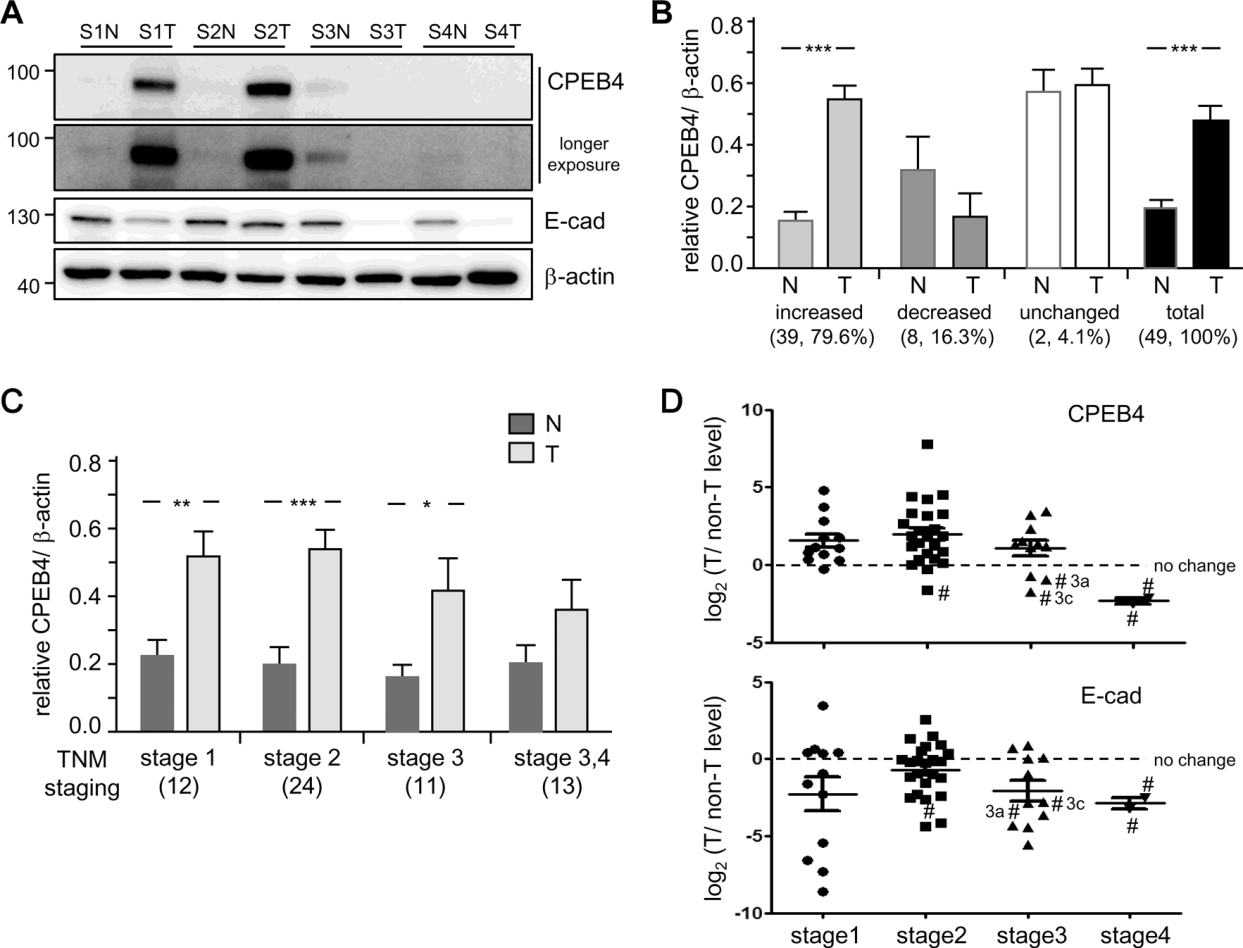
Increased CPEB4 protein level in most HCC specimens. Western blot analysis of CPEB4 MoAb (homemade#2) described in Fig 4A in tumorous (T) and adjacent non-tumorous (N) liver samples from 49 HCC patients at different stages (S1-S4). (A) Representative immunoblots of CPEB4, E-cadherin (E-cad) and the loading control β-actin in 4 paired HCC samples. After normalization to β-actin level, data from 49 paired samples were grouped by (B) CPEB4 level increased, decreased or unchanged in tumorous HCC samples or (C) HCC stage defined by the TNM system. (D) Fold change in CPEB4 and E-cad expression (T vs N) in each patient log2 transformed and plotted by tumor stage. Five of 8 CPEB4-downregulated samples with decreased expression of E-cadherin were denoted with number signs (#). Data are mean ± SEM. *P < 0.05, **P < 0.01, ***P < 0.001 by Student’s *t* test. Numbers in parentheses are number of samples.

Because we lack a CPEB4 antibody with good specificity for immunohistochemistry, we determined CPEB4 protein level in primary tumorous (T) and adjacent non-tumorous (N) liver tissues from 49 HCC patients by using immuoblotting with our CPEB4 monoclonal antibody (Fig 5A). Expression of E-cadherin (E-cad) and β-actin, a marker of epithelial cancers and a loading control, respectively, was also monitored. Downregulation of E-cadherin confers epithelial cells with enhanced metastatic and invasive potential (i.e., epithelial-mesenchymal transition) and is often found in malignant carcinomas [46]. We classified the level of normalized CPEB4 in paired liver samples from each HCC patient into three groups. Among the 49 paired samples, CPEB4 expression was upregulated in 39 (79.6%, N vs T: 0.17 ± 0.02 vs. 0.55 ± 0.04, *P* < 0.001), downregulated in 8 (16.3%, N vs T: 0.33 ± 0.09 vs. 0.18 ± 0.06, *P* = 0.21) and remained unchanged in 2 (4.1%) samples (Fig 5B). Data for all studied subjects are in Table 1. To analyze whether CPEB4 expression is associated with HCC malignancy, the same data were then classified by clinical tumor stage by the TNM system [29, 30]. Interestingly, CPEB4 upregulation was associated more with early-stage HCC (Fig 5C, N vs T: 0.22 ± 0.05 vs 0.52 ± 0.08 at stage 1; 0.20 ± 0.04 vs 0.54 ± 0.06 at stage 2) than late-stage HCC (N vs T: 0.16 ± 0.04 vs 0.42 ± 0.09 at stage 3; 0.20 ± 0.06 vs 0.37 ± 0.09 at stages 3–4). The fold change in CPEB4 and E-cadherin (E-cad) expression in T versus N tissue for each HCC patient after log2 transformation is in Fig 5D. Notably, 5 of 8 CPEB4-downregulated HCC samples were at stages 3 and 4. In addition, 5 of 8 CPEB4-downregulated samples also showed decreased expression of E-cadherin (marked with number sign # and in Table 1, highlighted in bold). The three samples with the most reduced expression of CPEB4 along with decreased E-cadherin level were from patients with very late-stage HCC (3c and 4). Most HCCs develop after chronic liver disease caused by hepatitis B virus (HBV) and/or HCV infection, and 42 HCC specimens were also HBV- and/or HCV-positive (Table 1). Nevertheless, we found no association of CPEB4 expression and other clinopathological factors such as age, sex or hepatitis viral infection (Table 1).

**Fig 6:**
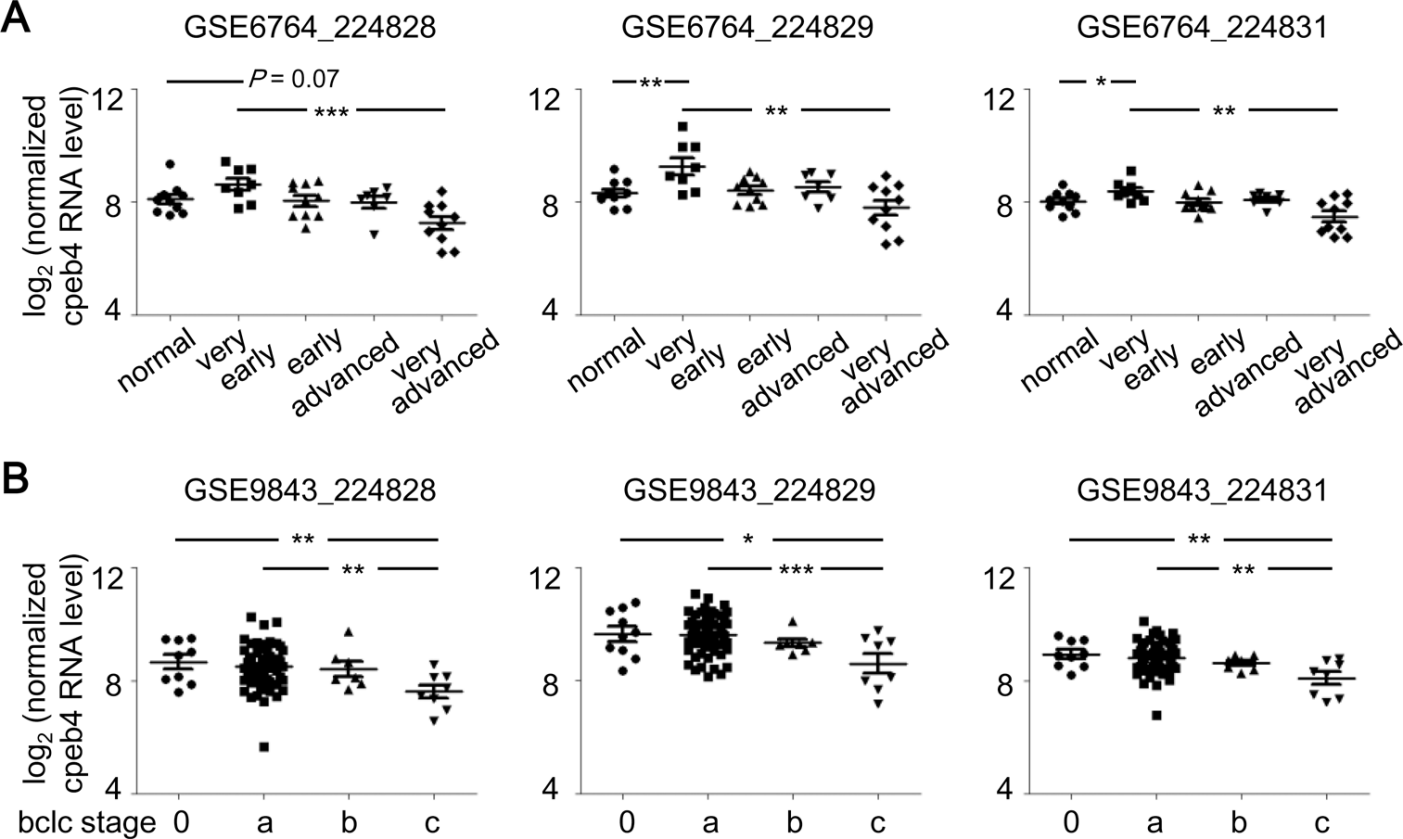
Bidirectional expression of CPEB4 mRNA associated with HCC stage. Two microarray datasets were downloaded from Gene Expression Omnibus. Normalized and log2-transformed CPEB4 RNA signals detected by the probes, 224828, 224829 and 224831, were extracted. (A) RT-PCR analysis of GSE6764, CPEB4 mRNA levels in normal liver tissues (n = 10) and very early (n = 8), early (n = 10), advanced (n = 7) and very advanced (n = 10) HCC specimens. (B) RT-PCR analysis of GSE9843, CPEB4 mRNA levels in HCC liver tissues staged by the Barcelona Clinic Liver Cancer (bclc) system, 0 (n = 9), a (n = 56), b (n = 7) and c (n = 8). Data are mean ± SEM. *P < 0.05, **P < 0.01, ***P < 0.001 by Student’s *t* test.

**Fig 7:**
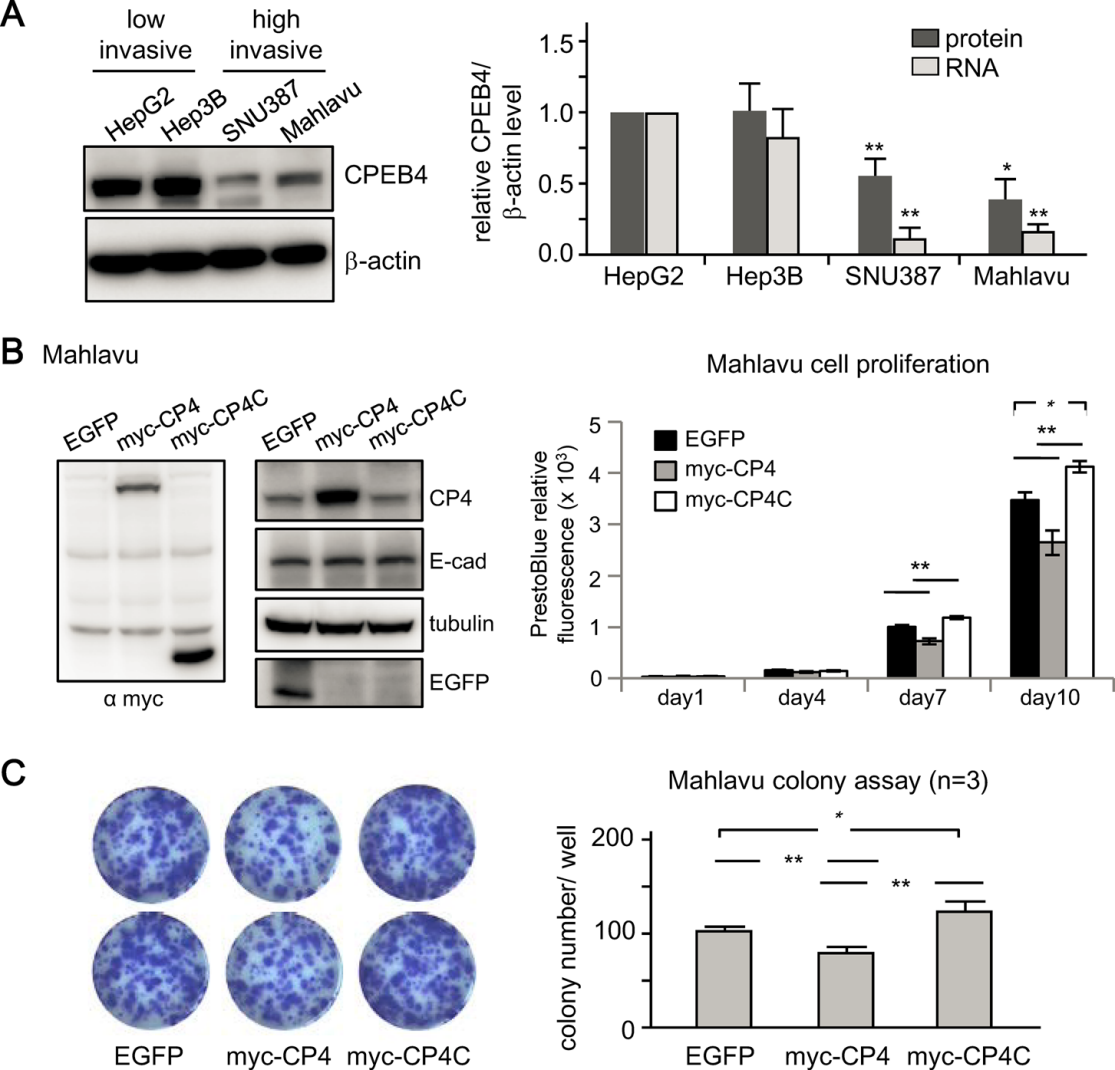
Expression of CPEB4 decreased colony formation of Mahlavu cells. (A) Protein and mRNA levels of CPEB4 in 4 HCC cell lines with differential invasive potential, HepG2, Hep3B, SNU387 and Mahlavu. Data are mean ± SEM from 3 independent experiments. *P < 0.05, **P < 0.01 compared with the corresponding expression level in HepG2 cells. Mahlavu cells infected with lentiviruses expressing EGFP, myc-tagged full length (myc-CP4) and C-terminus (myc-CP4C) of CPEB4 were used for (B) immunoblotting with the denoted antibodies and seeded at low density in 6-well plates. Cell proliferation was monitored at the indicated day with PrestoBlue live-cell labelling. (C) The cells grew for 10 days were fixed for the colony formation assay. Representative images are shown on the left. The number of colonies counted in wells from 3 independent experiments is expressed as mean ± SEM from 4 experiments. *P < 0.05, **P < 0.01 by Student’s *t* test.

Downregulation of CPEB4 in liver cancers appears to advance HCC progression only at a late stage. Nevertheless, we were unable to collect more specimens of stage 4 HCC because surgical resection with no obvious curative function is not recommended for patients with metastatic cancers. To further support our finding, we obtained two datasets from the GEO database, GSE6764 [34] and GSE9843 [35, 36], which contain transcriptome profiles from 45 and 80 liver specimens, respectively, isolated from normal liver and stage-diagnosed HCC liver. Both Affymatrix microarray datasets were analyzed for CPEB4 mRNA levels with the probe set (224828, 224829 and 224831). The GSE6764 dataset showed increased expression of CPEB4 mRNA in the very early stage of HCC (Fig 6A, 224829 and 224831 probes), with decreased CPEB4 mRNA level in the very advanced stage as compared with very early stage (Fig 6A). Similarly, in the GSE9843 dataset, significantly decreased CPEB4 mRNA level was associated with carcinogenic progression of HCC, defined by the Barcelona Clinic Liver Cancer (bclc) staging system [29, 30] (Fig 6B). Finally, using the cell lines established from well-differentiated HCC (i.e., HepG2 and Hep3B) and highly invasive HCC (i.e., SNU387 and Mahlavu), we found that CPEB4 protein and mRNA levels were decreased with cell malignancy (Fig 7A). To determine whether the increased expression of CPEB4 could reduce proliferation of Mahlavu cells, the cells infected with lentiviruses expressing EGFP, myc-tagged full length (myc-CP4) or C-terminal RNA-binding domain (myc-CP4C) of CPEB4 were used (Fig 7B). Elevated CPEB4 did not affect E-cadherin expression but reduced cell proliferation (Fig 7B) and colony formation (Fig 7C). In contrast, expression of myc-CP4C slightly increased proliferation (Fig 7B) and colony formation (Fig 7C) likely via competing with endogenous CPEB4 for binding to target RNAs but unable to regulate translation. Together with the CPEB4-KD results (Figs 2 and 3), alteration of CPEB4 expression may differentially affect growth of HCC cells depending on their degree of malignancy.

## Discussion

CPEB4 was first identified as a pro-oncogenic factor and promoted translation of tissue plasminogen activator (tPA) RNA to support metastatic invasion of pancreatic cancer cells [26]. After this study, CPEB4 expression was found upregulated in most glioma patients and inversely correlated with prognosis [47]. In contrast, CPEB4 expression was downregulated in 50% of 236 HCC cases and correlated with survival rate [27]. Here, we found that CPEB4 deficiency did not affect hepatic cell regeneration (Fig 1) but accelerated *in vitro* and *in vivo* growth of transformed HepG2 cells (Figs 2 and 3). CPEB4 expression was increased in 80% of 49 HCC patients but was decreased at the very late stage of HCC. This biphasic and stage-associated mRNA expression of CPEB4 in HCC was also documented in the GEO database. Thus, the role of CPEB4 in carcinogenesis may be more complicated. Depending on the cancer type and stage, CPEB4 may switch its role between oncogenic promoter and tumor suppressor.

Carcinogenesis from pre-neoplastic lesions to carcinomas requires stepwise changes in gene expression to transform epithelia to cancerous cells. Gene signatures closely associated with tumorigenic progression could be used as diagnostic markers and/or therapeutic targets. Thus, many efforts, such as the Cancer Genome Atlas and the Human Protein Cancer Atlas, were initiated for genome- and proteome-wide analyses of tumorous samples from various cancers. When perusing the literature, the specificity of CPEB4 antibodies used in previous studies raised our concern [27, 47, 48]. First, many commercial antibodies recognize CPEB4 at from 60 to 80 kDa by western blot analysis. Although the calculated molecular weight of CPEB4 (729 a.a.) is 80 kDa, CPEB4 migrates at about 90–95 kDa on a gel [26, 31]. Second, antibody non-specificity varies among tissues and species. An antibody working well in the mouse brain does not guarantee its specificity in other tissues or species (Fig 4). Third, CPEB4-immunoreactive bands of smaller size do not likely result from alternatively spliced *cpeb4* transcripts. The NCBI Reference Sequence Database contains five annotated human CPEB4 transcripts that encode CPEB4 of 729 (full-length), 712 (without exon 3), 704 (without exons 3 and 4), 339 and 332 amino acids. The *cpeb4* transcripts without exon 3 and/or 4 were also found in mice, but their translated products could not be separated from full-length CPEB4 [31]. Two shorter transcripts (339 and 322 a.a.) without exon 1 (375 a.a.) use the first methoine in exon 2 as the start codon. Nevertheless, we examined several commercial CPEB4 antibodies from Abcam, Gentex, Sigma and Santa Cruz Biotechnology and found the epitopes used to raise these antibodies are within the first 170 a.a. of exon 1, so immunodetected signals < 90 kDa result from antibody non-specificity (Fig 4). We could not find any CPEB4 antibody from Cell Signaling Technology, so we could not comment on the antibody specificity used in the glioma study [47]. Of note, CPEB2 (716 a.a) and human CPEB3 (684 and 698 a.a.) also migrate higher (~100 kDa) on a gel than their calculated molecular weights [18, 39]. The aberrant gel mobility of CPEBs2-4 is caused by their amino-terminal amino acid sequences and not by posttranslational modification, because *E*. *coli*-produced recombinant CPEB2-4 proteins also migrate at the same position with endogenous counterparts. Thus, specificity assessment of commercial CPEB antibodies is needed before using them in clinical specimens and determining the relation of CPEB expression in cancers.

Previous studies with microarray and chromatin-immunoprecipitation assays indicated that *cpeb4* was one of p53-transcribed genes in MCF7 breast cancer and U2OS osteosarcoma cells [49, 50]. Hep3B, SNU387 and Mahlavu cells express loss-of-function p53 mutants [51], but the CPEB4 RNA level in Hep3B cells is comparable to that in HepG2 cells expressing functional p53 (Fig 7A). Thus, despite p53 aberrations frequently being involved in HCC development [52, 53] and possibly contributing to CPEB4 downregulation in the late stage, transcription of *cpeb4* in HCC is not solely determined by p53.

CPEB4 likely plays versatile roles in carcinogenesis in a tissue- and stage-dependent manner. CPEB4 expression examined at the protein level (Fig 5) or mRNA level (Fig 6A and 6B) is significantly elevated in early-stage but decreased in late-stage HCC specimens. Because most liver samples in the GSE6764 [34] and GSE9843 [35, 36] studies were isolated from Caucasian patients, such a biphasic CPEB4 expression is not likely race-specific. Thus, contradictory findings between HCC studies of Chinese (50% showing decreased CPEB4 level by immunohistochemistry) [27] and Taiwanese (80% showing increased CPEB4 level by immunoblotting) may be due to antibody disparity. Notably, although CPEB4 is elevated in high-grade pancreatic intraepithelial neoplasia and differentiated PDA, it shows no significant change in level in the most malignant undifferentiated PDA [26]. Thus, stage-associated alteration in CPEB4 expression may also apply to other cancers besides HCC. We cannot comment on the study about CPEB4 expression in non-small cell lung cancer [54] because many figures in this paper were replicated from the HCC study [27]. Biphasic CPEB4 expression is closely associated with HCC staging. Although downregulation of CPEB4 appears to enhance tumorigenesis in late-stage HCC, a role for CPEB4 in early-stage HCC is unclear. Whether CPEB4 functions as an oncogenic promoter or suppressor in early-stage HCC and whether CPEB4 could be a diagnostic marker and/or therapeutic target in cancers need to be further investigated.

## Supporting Information

S1 Fig Representative images of control and CP4-KD HepG2 cells. HepG2 cells transfected with the plasmid expressing GFP and CP4-KD (siCP4) or control-KD (siCtrl) sequence were selected with G418 and collected for GFP-positive cells by a flow sorter.
(TIF)
