# Supplementary figures and images for: Biphasic and Stage-Associated Expression of CPEB4 in Hepatocellular Carcinoma
Source: PLoS One. 2016 May 9;11(5):e0155025. doi: 10.1371/journal.pone.0155025 (PMC4861299; doi:10.1371/journal.pone.0155025)

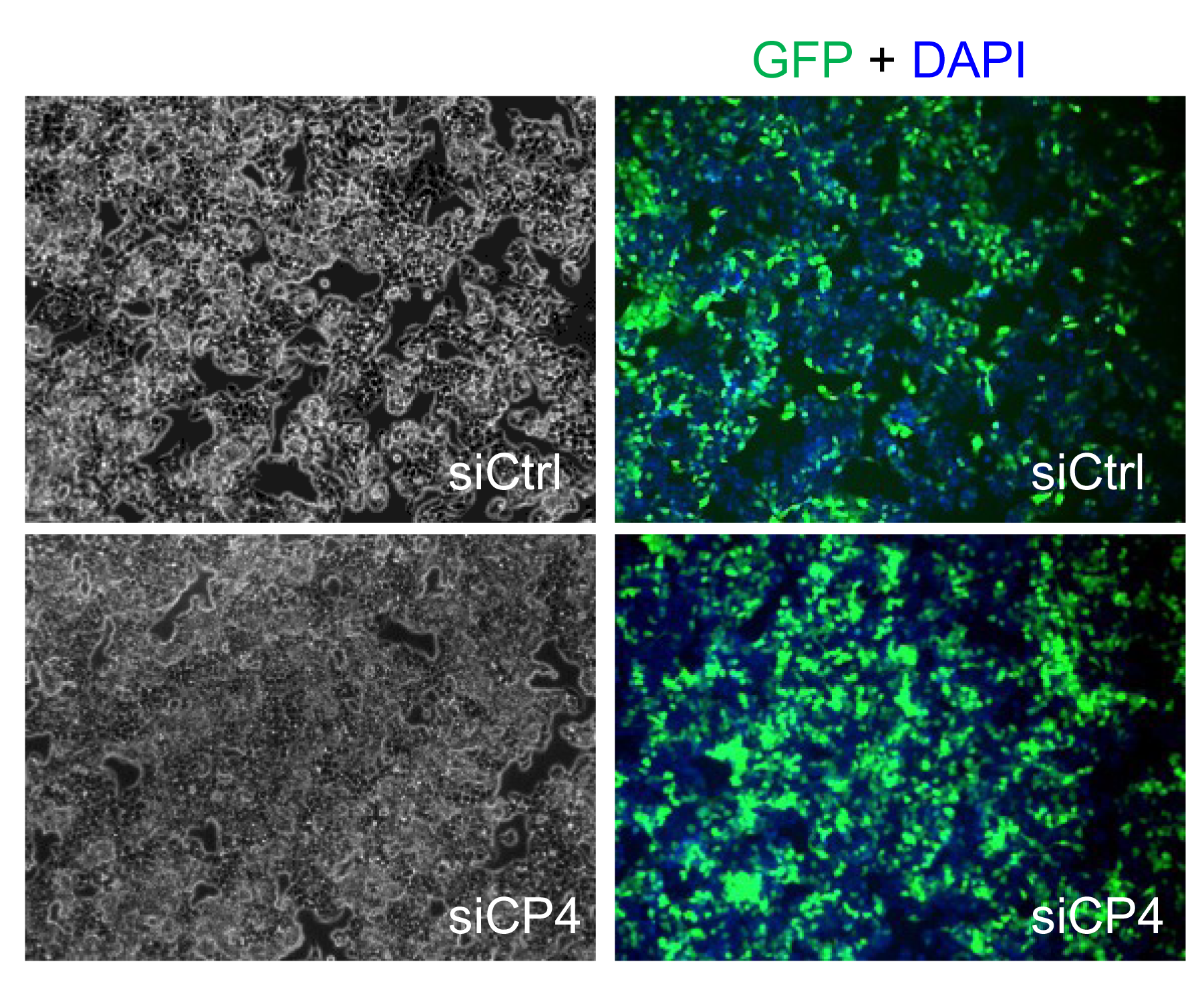

Supplement: S1 Fig — HepG2 cells transfected with the plasmid expressing GFP and CP4-KD (siCP4) or control-KD (siCtrl) sequence were selected with G418 and collected for GFP-positive cells by a flow sorter. (TIF) [file pone.0155025.s001.tif]
